# Human T Lymphotropic Virus Type I (HTLV-I) is a Risk Factor for Coronary Artery Disease

**Published:** 2013-03

**Authors:** Reza Farid Hosseni, Farahzad Jabbari, Mahmoud Shabestari, S. A. Rahim Rezaee, Yousef Gharivani, Narges Valizadeh, Mansoreh Sobhani, Toktam Moghiman, Farnaz Mozayani

**Affiliations:** 1 Allergy Research Centre; 2 Immunology Research Centre; 3 Preventive Cardiovascular Care Research Centre; 4 Inflammation Research Centre

**Keywords:** Coronary Artery Disease (CAD), HTLV-I, Risk factor

## Abstract

***Objective(s):*** Few studies have shown the association between HTLV-I infection and coronary artery disease (CAD). HTLV-I has been detected in heart autopsies, particularly in lymphoma\leukemia cases. Mashhad and Neyshabour (Razavi Khorasan Province, Iran) are endemic regions for HTLV-I. Therefore, the present study was carried out to evaluate the impact of HTLV-I on CAD in Neyshabourian patients.

***Materials and Methods:*** 7590 patients admitted to Razavi and Imam Reza Hospitals (2007-2008) were included in this study. The seroprevalance of HTLV-I infection was determined by the ELISA method and confirmed with the PCR method.

Statistical analyses were performed using the SPSS software.

***Results:*** Out of the 7590 studied subjects, 564 patients were born and had resided in Neyshabour. The HTLV-I sero-prevalence among these subjects was 13% (n=73). 294 subjects had an abnormal angiography (CAD) and among them 43 (14.6%) were sero-positive for HTLV-I. In the remaining 227 subjects who had a normal angiography, 30 cases were HTLV-I seropositve. The PCR test was performed on 35 cases in order to confirm the presence of infection, which was positive in 31. Regarding the initial population of 294, the rate of PCR-confirmed infection was 10.54%.

***Conclusion*** ***: ***This sero-prevalence of HTLV-I in subjects with heart complications in Neyshabour was nearly 3 times more than the general population of this city (10.5 % vs 3.4%). However, the results of this study show that in addition to HTLV-I infection, there might be other co-factors leading to the development of heart complications in Neyshabour.

## Introduction

Coronary artery diseases (CAD) cause 30% of all mortalities worldwide, making it the most common cause of death in men under the age of 65 and the second most common cause of death among women of the same age.

Atherosclerosis is an inflammatory disease with immunologic mechanisms accompanied by metabolic risk factors (1, 2); it is also the underlying etiology of 50% of coronary artery ectasia (CAE) cases with an incidence as high as 10% in some nations (3). In various studies the role of IL-1, TNF-α, IL-8, MCP-1a, IL-6, CRP and IFN-γ has been more prominent in determining the inflammatory symptoms of atherosclerosis (1, 2). In many cases with cardiovascular events, metabolic risk factors such as hyperlipidemia, hypercholesterolemia and diabetes have not been identified and the local or general inflammatory factors responsible for producing coronary artery disease have been introduced as chronic infection.

 The HTLV-I retrovirus, which was first diagnosed in 1980 in patients with skin lymphoma can lead to inflammatory disease and is endemic in the Caribbean region and South-West of Japan (4). Viral activity in the infected individual is very slow and most diseases related to this virus have a delayed appearance and manifest in the late stages of life.

The key point of infection with this virus is that more than 90% of the carriers remain asymptomatic whereas it may cause severe disorders such as ATL, HAM/TSP, HTLV-I infection-related dermatitis (IDH), uveitis, lymphocyte cell alveolitis, Sjogren’s syndrome, thyroiditis, Behcet disease, arthropathy and polymyositis in symptomatic patients. Moreover, this virus is endemic in the Khorasan region and based on the Farid *et al* study, the prevalence of HTLV-I in Mashhad and Neyshabour (Razavi Khorasan, Iran) was 2.3% and 3.4%, respectively. According to another study performed in 2003 by Abbaszadegan *et al* in Mashhad on 28926 blood donors (81.8% male and 18.14% female), the prevalence of HTLV-I infection was 0.78% (5-9).

Among the diseases related to HTLV-I, extensive studies have been performed on the pathogenesis of HAM/TSP and various reports have shown that HTLV-I infection in such cases is accompanied by the spontaneous proliferation of T-cells and the production of high levels of pro-inflammatory cytokines such as IL-2, IL-4, IL-6, TNF-α, IL-1α, IL-1β, IFN-γ in serum and CSF and spinal cord injuries in infected patients (4,5,7-11).

 In the latest review article by Dr. Gessain from the French Pasteur Institute, it has been stated that the main etiology of HTLV-I related diseases might be cardiovascular complications. Therefore, regarding the cardiovascular nature of HTLV-I and the role of inflammation in the pathogenesis of atherosclerosis and coronary artery disease (CAD) and the endemicity of Neyshabour region for HTLV-I infection; in this study the rate of HTLV-I infection and its possible inflammatory effects in patients with coronary vessel involvement was investigated in order to evaluate its risk ratio in causing cardiac disease.

## Materials and Method

Considering the outbreaks of HTLV-I infection in Neyshabour, in a retrospective study, 7590 patients with cardiac symptoms who had undergone angiography in Imam Reza and Razavi hospitals of Mashhad, were enrolled from March 2007 to April 2008. The records of patients from Neyshabour were then studied and those with a positive HTLV-I test before angiography, performed by the ELISA method using an available commercial kit (Dia-Pro, Italy), were determined.

For confirming the presence of infection in patients with HTLV-I positive serology tests, after gaining an informed consent, a 5 cc blood sample was obtained from the brachial vein in order to perform the PCR test.

Genomic DNA was extracted from peripheral blood mononuclear cells (PBMC) using an available commercial kit (Blood mini kit, Qiagen, Germany) and PCR amplification was performed using specific primers for tax (5_- AGGGTTTGGACAGAGTCTT-3_ and 5_-AAGGACCTTGAGGGTCTTA- 3_) and LTR regions (5_-CATAAGCTCAGACCTCCGGG-3_ and 5_- GGATGGCGGCCTCAGGTAGG-3_).

***Statistical methods***

The collected data were initially entered into the SPSS software, version 16.0. They were then described by tables and statistical indexes. In the end, ANOVA, T-test and Chi-square test were applied in order to analyze the relationship between the studied variables.

## Results

In this study, 564 patients (M=314, F=379) with cardiac symptoms from the endemic region of Neyshabour, which has a prevalence rate of 3% for HTLV-I infection in the general population, were assessed. 73 cases (13%) had a positive serologic test for HTLV-I by the ELISA method.

The average age of the HTLV-I infected patients was 61.1 years and the average age of the non-infected group was 58.17 years. According to the independent sample test, no significant difference was observed regarding age (*P=*0.045).

In the HTLV-I infected group the average age of the male population was 61 years, whereas it was 61.3 years among females; again showing no statistically meaningful difference regarding sex (*P=*0.824).

In total, 227 cases had a normal angiography, whereas 294 had coronary artery involvement (one to three vessels). The number of subjects with HTLV-I infection among Neyshabourian patients with normal coronary arteries was 30 and in those with coronary artery involvement, it was 43 (14.6%). Based on the Chi-square test no significant difference was revealed in the prevalence of HTLV-I between genders among the cases with coronary artery involvement (*P=*0.081).

In patients with coronary artery disease and a positive HTLV-I test by the ELISA method, the PCR test was performed on 35 cases in order to confirm the presence of infection, which was positive in 31. Regarding the initial population of 294 cases, the rate of PCR-confirmed infection was 10.54%.

## Discussion

Few studies have shown cardiac involvement in HTLV-I infection. The first written report on the probable prevalence of HTLV-I in Khorasan was published in 1993 in the Leukemia Journal. It described the cardiac involvement of one patient from Mashhad diagnosed by Gabere in France. The patient was a 60-year-old lady from Mashhad whom had undergone valve replacement surgery due to mitral and aortic valve impairment. Through a histopathological study of the valves, non-hodgkin T-cell lymphoma, HTLV-I antibody in serum and on the DNA of valve cells, peripheral blood mononuclear cells (PBMCs) and specific env HTLV-I with the PCR method were detected (12).

In this patient, ATL was confirmed as the chronic form with slow progression. This was the first report of isolated lymphoma involvement of the cardiac valve without any other cardiac abnormalities.

In 1997 a report on cardiac autopsy in three cases with lymphoma/leukemia was published in the Leukemia Journal. In this study which discussed two cases of ATL and one case of promyelocytic leukemia, cardiac involvement ranging from microscopic to macroscopic infiltration similar to myocardial infarction and myocardial microabscess was mentioned (13). Connolly *et al*, in a case report in 2002 described a 61-year-old African male from the Caribbean Islands with bowel obstruction and intestinal lymphadenopathy. This patient suffered from non-Hodgkin T-cell lymphoma accompanied by HTLV-I infection. From the clinical study, tachypnea and tachycardia were diagnosed. From echocardiography, a mass in the right atrium originating from the interatrial septum and another mass in the lateral wall of the right atrium protruding into the left ventricle was observed. Cardiac involvement has been repeatedly reported in lymphoma cases. Intra cardiac involvement of non Hodgkin T-cell lymphoma, which responds well to treatment, has only been seen in AIDS patients. This is the first report of cardiac involvement in lymphoma alongside HTLV-I infection (14).

In the Kumamoto *et al* study, a 56-year-old male infected with HTLV-I resulting in multi-organ failure and calcification of systemic organs on autopsy, was reported. Advanced heart failure in addition to lung and kidney failure, HTLV-I antibody in serum and increased calcium and parathyroid hormone levels were diagnosed in this patient (15).

In cases of HTLV-I infection accompanied by ATL, malignant involvement of the heart is rarely seen. In the O’Mahony *et al* study, 8 patients with ATL and confirmed pathological findings of cardiac involvement were reported. Pericardial infusion and tamponade, cardiac valve infiltration, valvular nodular tumor and myocardial and endocardial tumors were observed in these patients (16).

Cardiac involvement in ATL is commonly confirmed after death whereas in the mentioned study the three cases were clinically diagnosed during their lifetime. In this study, in all patients except one, the ATL was of the acute progressive type and in addition to cardiac symptoms, they also suffered from pulmonary symptoms. One case of chronic ATL survived for 10 years after performing the valve replacement surgery (16).

Iemura *et al* reported an ATL case with extensive cardiac involvement, which was initially presented with symptoms of fever and cervical lymphadenopathy. He had responded to immunosuppressive therapy, but in the second visit he had experienced palpitation and dyspnea and consequently died due to heart failure.

In the autopsy done for this patient, the heart was severely enlarged and the myocardium was substituted with atypical lymphoid cells.

Cardiac involvement in ATL is mainly due to tumor expansion or the cardiac lymphatic back flow from the mediastinal lymphatic system (17).

In 2001 Thiam described a case of a 17-year-old African girl from Senegal with pericardial infusion, which was later diagnosed as left ventricular non-Hodgkin lymphoma. Both HTLV-I and HIV were positive in this patient (18).

Stuver studied HTLV-I related diseases among 1834 Japanese cases in 1996. He found a relationship between HTLV-I and asthma (relative risk=3.4) and also unexpectedly another correlation with a history of cardiac disease (relative risk=1.4). Moreover, Masuda reported a 16% prevalence rate for HTLV-I among the 517 patients for which he performed on-pump cardiovascular surgery (19).

Finally, although this therapeutic method did not show any significant difference between the two groups with or without HTLV-I infection, but the rate of post-surgical infections was remarkably higher in cases positive for HTLV-I infection. Therefore, this virus could be considered as one of the risk factors leading to such complications (relative risk=2.4%).

## Conclusion

The sero-prevalence of HTLV-I infection in subjects with heart complications in Neyshabour was nearly 3 times more than the general population of this city (10.5 % vs. 3.4%). The probable correlation between HTLV-I infection and coronary vessel involvement can be introduced as a risk factor for coronary artery disease with the mechanism of vascular inflammation.

## References

[1] Kuznetsov GP, Lebedev PA (1995). The clinical significance of selenium deficiency in patients from the Samara region with cardiovascular diseases and its correction with the preparation Selena. Eksp Klin Farmakol.

[2] Hassanzadeh M, Faridhosseini R, Mahini M, Faridhosseini F, Ranjbar A (2006). Serum Levels of TNF-Alpha, IL-6, and Selenium in Patients with Acute and Chronic Coronary Artery Disease. Iran J Immunol.

[3] Shabestari MM, Jabbari F, Gohari B, Moazen N, Azizi H, Moghiman T, Ibrahimzadeh S, Amirabadi A (2011). Coronary artery angiographic changes in veterans poisoned by mustard gas. Cardiology.

[4] Farid R, Parizadeh MJ, Ghaffari J, Miri S, Nasirian A, Rafatpanah H (1383). Seroepidemiologic study of HTLV-I in Neyshabour. Mashhad Med Faculty J.

[5] Rafatpanah H, Pravica V, Faridhosseini R, Tabatabaei A, llier W, Poulton K, Thomson W, Hutchinson I (2007). Association between HLA-DRB1*01 and HLA-Cw*08 and outcome following HTLV-I infection. Iran J Immunol.

[6] Rafatpanah H, Pravica V, Farid R, Abbaszadegan MR, Tabatabaei A, Goharjoo A (2004). Hum Immunol.

[7] Farid R, Pishnamaz R (1381). HTLV-I infection and accompanying diseases. Mashhad Med Faculty J.

[8] Fukushima N, Nishiura Y, Nakamura T, Kohno S, Eguchi K (2007). Blockade of IL-2 receptor suppresses HTLV-I and IFN-gamma expression in patients with HTLV-I-associated myelopathy/tropical spastic paraparesis. Int ED.

[9] Goon PK, Igakura T, Hanon E, Mosley AJ, Asquith B, Gould KG, Taylor GP, Weber JN, Bangham CR (2003). High circulating frequencies of tumor necrosis factor alpha-and interleukin-2-secreting human T-lymphotropic virus type 1(HTLV-I)-Specific CD4+ T cells in patients with HTLV-I Associated neurological disease. J Virol.

[10] Best I, Adaui V, Verdonck K, Gonzalez E, Tipismana M, Clark D, Gutuzzo E, Vanham G (2006). Proviral load and immune markers associated with human T-Lymphotropic virus type 1 (HTLV-I)- associated myelopathy/tropical spastic paraparesis (HAM/TSP) in peru. Clin Exp Immunol.

[11] Santos SB, Porto AF, Muniz AL, de Jesus AR, Magalhaes E, Melo A (2004). Exacerbated inflammatory cellular immune response characteristics of HAM/TSP is observed in a large proportion of HTLV-I asymptomatic carriers. BMC Infect Dis.

[12] Gabarre J, Gessain A, Raphael M, Merle-Béral H, Dubourg O, Fourcade C (1993). Adult T-cell leukemia/lymphoma revealed by a surgically cured cardiac valve lymphomatous involvement in an Iranian woman: clinical, immunopathological and viromolecular studies, Leukemia.

[13] Daisley H, Charles W (1997). Cardiac involvement with lymphoma/leukemia: a report of three autopsy cases. Leukemia.

[14] A Hamaad, R C Davis, D L Connolly (2002). Regression of HTLV-I associated intracardiac lymphoma following chemotherapy. Heart.

[15] Kumamoto H, Ichinohasama R, Sawai T, Naganuma H, Furukawa Y, Akiu N (1998). Multiple organ failure associated with extensive metastatic calcification in a patient with an intermediate state of human T lymphotropic virus type I (HTLV-I) infection: report of an autopsy case. Pathol Int.

[16] O’Mahony D, Debnath I, Janik J, Aisner D, Jaffe E, Waldmann T (2008). Cardiac involvement with human T-cell lymphotrophic virus type-1-associated adult T-cell leukemia/lymphoma: The NIH experience. Leuk Lymphoma.

[17] Iemura A, Yano H, Kojiro M, Nouno R, Kouno K (1991). Massive cardiac involvement of adult T-cell leukemia/lymphoma. An autopsy case. Arch Pathol Lab Med.

[18] Thiam M, Dangou JM, Poplin S, Klotz F, Perret JL (2001). Cardiac T cell lymphoma: a case report. Dakar Med.

[19] Stuver SO, Tachibana N, Okayama A, Mueller NE (1996). Evaluation of morbidity among human T lymphotropic virus type 1 carriers in Miyazaki. Japan J Infect.

